# Genetic Control of Courtship Behavior in the Housefly: Evidence for a Conserved Bifurcation of the Sex-Determining Pathway

**DOI:** 10.1371/journal.pone.0062476

**Published:** 2013-04-22

**Authors:** Nicole Meier, Simone Catherine Käppeli, Monika Hediger Niessen, Jean-Christophe Billeter, Stephen F. Goodwin, Daniel Bopp

**Affiliations:** 1 Institute of Molecular Life Sciences, University of Zürich, Zürich, Switzerland; 2 Center for Behavior and Neurosciences, University of Groningen, Groningen, The Netherlands; 3 Department of Physiology, Anatomy and Genetics, University of Oxford, Oxford, United Kingdom; University of Arkansas, United States of America

## Abstract

In *Drosophila melanogaster*, genes of the sex-determination hierarchy orchestrate the development and differentiation of sex-specific tissues, establishing sex-specific physiology and neural circuitry. One of these sex-determination genes, *fruitless* (*fru*), plays a key role in the formation of neural circuits underlying *Drosophila* male courtship behavior. Conservation of *fru* gene structure and sex-specific expression has been found in several insect orders, though it is still to be determined whether a male courtship role for the gene is employed in these species due to the lack of mutants and homologous experimental evidence. We have isolated the *fru* ortholog (*Md-fru*) from the common housefly, *Musca domestica*, and show the gene’s conserved genomic structure. We demonstrate that male-specific *Md-fru* transcripts arise by conserved mechanisms of sex-specific splicing. Here we show that *Md-fru*, is similarly involved in controlling male courtship behavior. A male courtship behavioral function for *Md-fru* was revealed by the behavioral and neuroanatomical analyses of a hypomorphic allele, *Md-tra^man^*, which specifically disrupted the expression of *Md-fru* in males, leading to severely impaired male courtship behavior. In line with a role in nervous system development, we found that expression of *Md-fru* was confined to neural tissues in the brain, most prominently in optic neuropil and in peripheral sensory organs. We propose that, like in *Drosophila*, overt sexual differentiation of the housefly depends on a sex-determining pathway that bifurcates downstream of the *Md-tra* gene to coordinate dimorphic development of non-neuronal tissues mediated by *Md-dsx* with that of neuronal tissues largely mediated by *Md-fru*.

## Introduction

The genetic pathway controlling sex-specific behaviors in the invertebrate model system *Drosophila melanogaster* has been elucidated in great detail [1], [2]. These studies revealed that the genetic control of male mating behavior in *Drosophila melanogaster* is largely governed by the transcription factor *fruitless* (*fru*) [3], [4]. This gene acts as a pivotal switch for the correct developmental implementation of male-specific behaviors. A specific class of *fru* alleles have been identified which impairs courtship performance of males, but leaves females unaffected [5], [6], [7]. Moreover, it was demonstrated that expression of the male-specific *fru* functions in genotypically female flies elicits some aspects of male courtship behavior [8], [9], [10], [11]. However, the conclusion that *fru* is not only necessary but also sufficient for implementing male mating behavior in *Drosophila* has been challenged in recent studies [12], [13], [14], [15], [16]. These studies suggest that the sex-determining double-switch gene *doublesex* (*dsx*) also contributes to the regulation of male mating behavior.

The *fru* gene in *Drosophila melanogaster* is very large in size spanning approximately 150 kb of genomic sequences. Transcription initiates from four independent promoters (P1–P4) and the resulting transcripts undergo a complex pattern of alternative splicing events at both the 5′ and the 3′ ends, generating 18 different transcript variants which code for a family of transcription factors containing a common BTB domain and one of four alternative zinc-finger DNA binding domains [17]. The distal most promoter P1 gives rise to transcripts, which are implicated in regulating sex-specific behaviors [3], [4]. Transcripts derived from the other three promoters are non sex-specific and have vital developmental functions in both males and females [18]. Sex-specific expression is achieved by alternative splicing at the 5′ end of P1 transcripts mediated by the splice regulator *transformer* (*tra*), a key component of the sex-determining pathway. In females, TRA protein forms a splice enhancer complex with TRA2 and RBP1 (RNA Binding Protein 1) binding to nascent *fru* RNAs where they act to alter splice site choice, resulting in the inclusion of a pre-mature stop codon signal and thereby generating female-specific transcripts (*fru^F^*) with a severely truncated ORF [3], [19]. In males, where the *tra* gene is functionally OFF, the default splice-site is used and the resulting male-specific transcripts (*fru^M^*) encode functional full-length BTB-Zn-finger type transcription factors. These sex-specific transcripts incorporate alternate 3′ exons A, B, or C to encode the Fru^M^ isoforms: Fru^MA^, Fru^MB^ and Fru^MC^. Fru^M^ isoforms are translated only in males and are expressed in all regions of the nervous system implicated in male courtship behavior [12], [20], [21], [22], [23]. These FRU^M^ positive neurons located in widely distributed clusters throughout the male brain and nerve chord are key parts of the neuronal circuitry required for behavioral responses to sex pheromones and the display of proper male courtship behavior.

Given the critical role of *fru* in controlling sex-specific behaviors the question arises whether this function is conserved in other insects. The existence of sex-specifically expressed *fru* homologs in various dipteran and in hymenopteran species (*Anopheles gambiae* [24], *Aedes aegypti* [25], *Ceratitis capitata* [26] and *Nasonia vitripennis* [27]) suggests that *fru* has a conserved male-specific function in holometabolous insects. However, functional studies have yet to establish a definitive role in male courtship behavior in these species.

In our study we used the common housefly, *Musca domestica*, as an experimental system to investigate the genetic control underlying male courtship behavior. In previous studies we showed that all aspects of sexually dimorphic development including behavior are governed by a key switch gene, previously referred to as *F* and more recently identified as an ortholog of the *transformer* gene, *Md-tra* [28], [29]. A male-determining *M-*factor, which is located on the Y chromosome in standard strains but can also be found on autosomes in natural populations, serves as the primary instructive signal [29]. When *M* is absent, zygotic *Md-tra* is spliced in a mode that generates transcripts with an intact ORF. These *Md-tra* messages code for a RS-type of splicing regulator, which through an autocatalytic function upholds the productive splicing of its own pre-mRNA. Maternally deployed *Md-tra* activity is required to engage this positive feedback loop in the early zygote [28]. Presence of *M* in the zygote invokes a change in splicing of *Md-tra,* which results in the production of messages with a truncated ORF. Likewise, a previously identified target of *Md-tra*, *the Musca doublesex* homologue, *Md-dsx*, is regulated by sex-specific processing of its pre-mRNA [30]. Md-TRA together with its co-factor Md-TRA2 directs the female-specific splice mode of *Md-dsx* generating a female-specific variant which instructs female differentiation [31]. In *M* containing individuals, where *Md-tra* remains OFF, the male-specific splice mode of *Md-dsx* is established by default and a male-specific isoform is generated thereby ensuring male development.

Here we report the identification of the housefly ortholog of *fruitless*, *Md-fru,* and show that this gene is a downstream target of *Md-tra.* In an analogous way to *Drosophila*, *Md-fru* expresses functional splice variants only in males where *Md-tra* is OFF. Residual levels of *Md-tra* activity in males homozygous for the hypomorphic *Md-tra^man^* allele, prevents expression of functional *Md-fru* products leading to sterility. Our findings suggest that the correct implementation of male mating behavior is governed by a specific branch of the sex-determining pathway mediated by *fru* in both *Musca* and *Drosophila*.

## Results

### *Md-fru* is Sex-specifically Regulated and a Target of *Md-tra*

**Figure 1:**
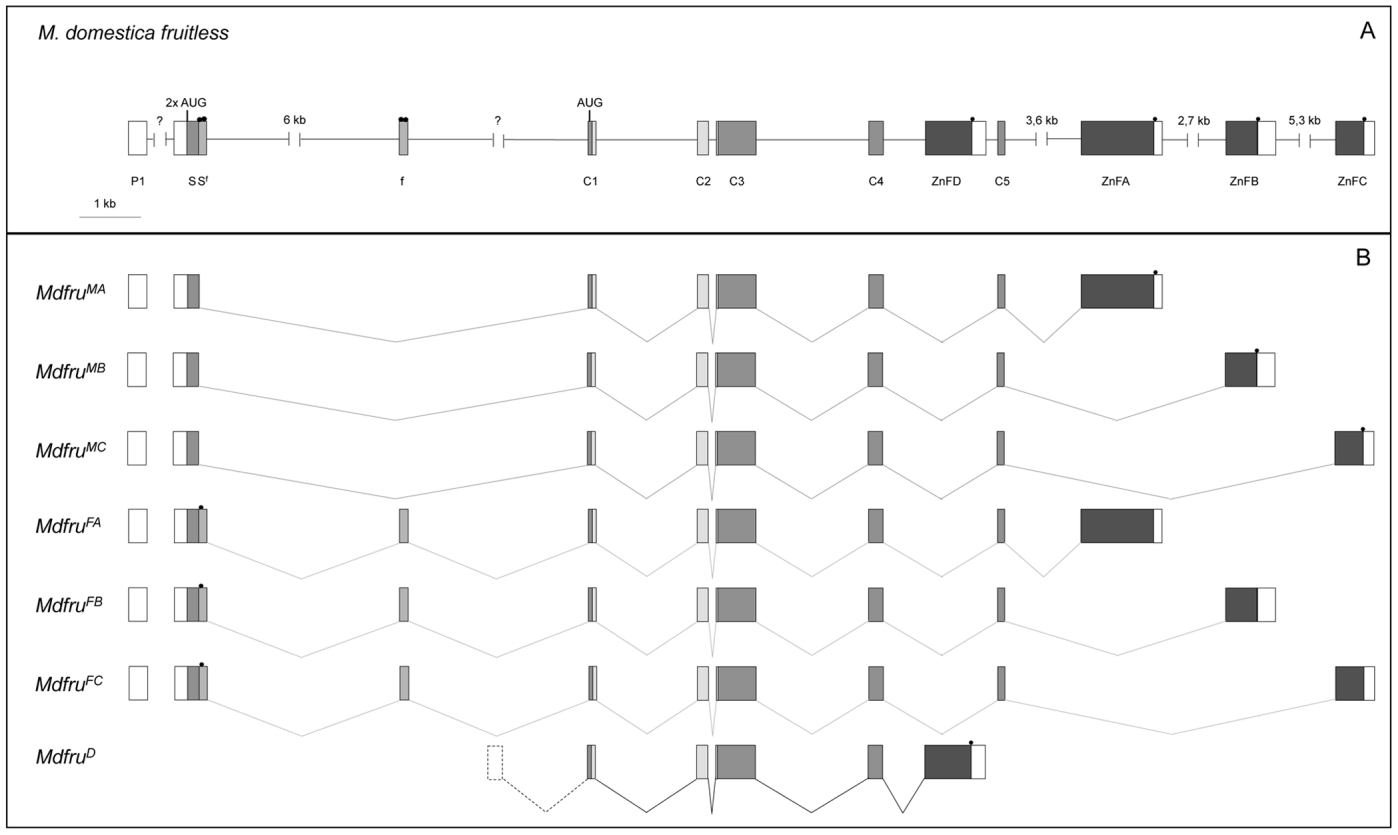
Genomic organisation and multiple transcript variants of *Md-fru*. (A) Schematic drawing of the *Md-fru* locus. Coding exons are indicated as grey shaded boxes, while 5′ and 3′ UTRs are shown as white boxes. Sequences in C1, C2 and C3 exons encoding the BTB domain are labelled in light grey and exons encoding the zinc finger-like domains (ZnFA, ZnFB, ZnFC, ZnFD) are shown in dark grey. P1 labels the most distally identified exon followed by the common exon S, and the two female-specific exons Sf and f. Small black dots mark the positions of in-frame stop codons (two in each female-specific exon, one in each zinc finger encoding exon). Two potential translational start codons are present in the exon S, and one in exon C1. (B) Multiple transcript variants identified by RT-PCR analysis in male and female head RNA samples. In male samples, three variants were detected (*Md-fru^MA^ Md-fru^MB^* and *Md-fru^MC^*) each of which includes a different zinc finger exon. None of them include the female-specific exons Sf and f and. The splice variants *Md-fru^FA^ Md-fru^FB^* and *Md-fru^FC^* were only detected in female head samples, all included the female-specific exons Sf and/or f which introduce several in-frame translational stop codons that prematurely terminate the ORF. In addition, we identified a nonsex-specific transcript variant (*Md-fru^D^*) which incorporates exon ZnFD but does not originate from P1. Preliminary results from 5′ RACE studies indicate that the 5′ end of this transcript is located in the intron sequences between exons f and C1 and thus may derive from a different promoter.

In *Drosophila*, the pathway downstream of *tra* bifurcates and employs the gene *fruitless* (*fru*) to control various aspects of sexually dimorphic CNS development [18], [32]. To establish if *Md-tra* also regulates housefly courtship through a *fru*-like downstream effector we set out to isolate the *fru* ortholog in Musca. PCR amplification with degenerate primers flanking the conserved BTB domain of *fru* yielded a genomic fragment of 190 bp in length. Subsequent extension by 3′ RACE of cDNAs prepared from adult houseflies revealed the existence of four alternative mRNA transcripts (Fig. 1). All 4 variants were present in female and male cDNA preparations and each encoded a different zinc finger type motif at its carboxy-terminal end. The same complex pattern of 3′ end processing has been reported in *fru* orthologs of *Drosophila melanogaster*, *Anopheles gambiae*, *Aedes aegypti*, *Nasonia vitripennis*, and *Tribolium castaneum*, [24], [25], [27]; Nicole Meier and Daniel Bopp, unpublished results). Sequence alignments of the differentially processed exons showed a high degree of similarity to the corresponding *Drosophila* exons (Fig. S1), so the genomic order of the differentially spliced 3′ exons is additionally conserved.

**Figure 2:**
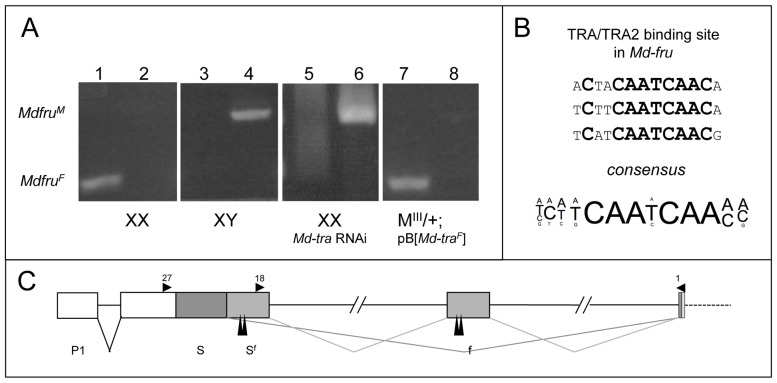
Sex-specific splicing of *Md-fru* is mediated by *Md-tra*. (A) RT-PCR results with sex-specific sets of *Md-fru* primer pairs. Amplification with *Md-fru^F^* specific primers Md-fru-18 and Md-fru-1 in wildtype XX females (1), in wildtype XY males (3), in *Md-tra* silenced XX females (5) and in ectopically *Md-tra^F^* expressing *M^III^* males (7). Amplification with *Md-fru^M^* specific primers Md-fru-27 and Md-fru-1 in wildtype XX females (2), in wildtype XY males (4), in *Md-tra* silenced XX females (6) and in ectopically *Md-tra^F^* expressing *M^III^* males (8). In wildtype XX flies, where *Md-tra* is active, only *Md-fru^F^* but no *Md-fru^M^* transcripts can be detected (1 and 2). A clear shift to *Md-fru^M^* splicing can be observed in XX individuals when *Md-tra* is silenced (5 and 6). Likewise, only *Md-fru^M^* transcripts are present in wildtype XY males, where *Md-tra* is not active (3 and 4). *Md-fru* splicing shifts to the female mode in *M^III^* males when continuous *Md-tra^F^* activity is provided by a transgene [28] (7+8). (B) Direct splicing regulation by *Md-tra* is suggested by the presence of putative TRA/TRA2 binding sites in *Md-fru*. Comparison of three different sequences found in *Md-fru* with the TRA/TRA2 binding site consensus derived from sequence alignments of binding sites found in the *fru* and *dsx* orthologs of *Drosophila melanogaster*, *Musca, domestica* and *Anopheles gambiae.* (C) Putative TRA/TRA2 binding sites (long vertical arrowheads) are found in close vicinity to the regulated splicing sites in the *Md-fru* locus. Positions of the primers used in this expression analysis are indicated by small horizontal arrowheads.

In *Drosophila*, the genes acting immediately before *fru* in the sex determination hierarchy direct sex-specific splicing of transcripts derived from the most distally located P1 *fru* promoter [3], [19]. To examine the corresponding upstream sequences of the putative housefly *fru* ortholog, 5′ RACE was performed on cDNA templates prepared from RNA extracted from adult male and female heads. In male preparations, we detected transcripts that contained two additional exons (P1 and S) upstream of the BTB coding exons (C1–C3) extending the ORF by an additional 72 amino acids at the amino terminal end (Figs. 1, S1). In female transcripts we observed that exon S was significantly larger in size due to the utilization of a more downstream located splice donor site (S^f^). In addition, we identified a female-specific exon (f) downstream of exon S^f^ (Fig. 1). These additional exonic sequences, which are absent in male transcripts, contain several in-frame stop codons, which prematurely truncate the long ORF as defined by the male transcripts (dots in Fig. 1A). Four repeat elements were identified in these female-specific sequences that perfectly match the *cis*-regulatory elements (TRA/TRA2 binding sites) known to play an important role in sex-specific splicing of *Drosophila fru* (Fig. 2). The male-specific transcripts encode full-length products, which show a high degree of protein sequence similarity to those of P1-derived male transcripts in *Drosophila* and are therefore referred to as *Md-fru^M^* (GenBank Accession Number KC750909, KC750911 and KC750913). The female-specific transcripts, on the other hand, have a severely truncated ORF and thus are likely to represent non-functional splice variants of the same locus. Because of their structural correspondence to the non-functional products of P1-derived female transcripts in *Drosophila* they are collectively named *Md-fru^F^* (GenBank Accession Number KC750910, KC750912 and KC750914). Taken together we conclude that the *Musca* genome harbors an ortholog of *fru, Md-fru,* which expresses a set of male-specific products and hence is likely to be a target of the sex determining pathway.

The presence of conserved TRA/TRA2 binding sites in *Md-fru,* suggested that it is sex-specifically spliced and directly targeted like *Md-dsx*, by *Md-tra*. To explore this possibility, we tested expression of *Md-fru* in different genetic backgrounds in which *Md-tra* activity was experimentally altered (Fig. 2). Previous studies demonstrated that silencing by early dsRNA injections is an effective means to deplete *Mdtra* activity in XX embryos, resulting in a complete shift of *Md-dsx* splicing from female to male mode [28]. Likewise, we observed that the splicing mode of *Md-fru* changed from female to male in *Md-tra* silenced XX individuals (Fig. 2A). The same result was obtained when *Md-tra2*, an essential co-factor of *Md-tra*, was depleted by RNAi (data not shown). We conclude that female splicing of *Md-fru* depends on the presence of *Md-tra* and *Md-tra2* activity supporting our notion that *Md-fru* is a downstream target of *Md-tra* in the sex determination pathway. To extend this type of epistatic analysis we investigated whether ectopic expression of the female activity of *Md-tra* is sufficient to direct female-specific splicing of *Md-fru* in genotypic males. To this end, we used a transgenic line in which *Md-tra^F^* is constitutively expressed in flies carrying the male determining factor *M^III^* on the third chromosome [28]. These individuals develop into fully fertile females demonstrating that forced *Md-tra^F^* expression overrides repression by *M^III^* and is sufficient to direct all aspects of female development. RNA samples analyzed from the heads of these sex-reverted *M^III^/+*; p[*Md-tra^F^*] individuals showed the presence of only female-specific splice variants of *Md-fru* (Fig. 2A). Hence, *Md-tra* activity is not only required but also sufficient to impose female splicing mode of *Md-fru*. The presence of well-conserved TRA/TRA2 binding sites around the critically involved splice sites suggested that *Md-fru* is a direct target of *Md-tra* (Fig. 2B,C).

### *Md-tra^man^* Males Express Female *Md-fru* Transcripts and are Impaired in Courtship

We previously described the *masculinizer* (*man*) allele of *Md-tra* to be a hypomorphic mutation with residual *Md-tra* activity [28], [33]. Flies homozygous for *Md-tra^man^* develop into phenotypically normal looking males (from here on referred to as *Md-tra^man1^* males). However, they are reduced in fertility, as only a fraction (about 10%) produces progeny in single mating tests. Sterility is 100% in homozygous *Md-tra^man^* males when their mother carried the dominant gain-of-function allele *Md-tra^D^* in trans [28], [34], [35]. Males with this maternal background were referred to as *Md-tra^man2^* males. The genital apparatus of *Md-tra^man1^* and *Md-tra^man2^* males looked indistinguishable in morphology from those of wildtype males, and normal amounts of motile sperm were present in squashed preparations of mutant testes. To test for gonad and sperm functionality, the larval anlagen of *Md-tra^man2^* testes were transplanted into wildtype male hosts (Fig. S2). As these mosaic animals were capable of siring a large number of donor-derived offspring, we concluded that sperm maturation was normal in *Md-tra^man2^* mutant testes.

**Figure 3:**
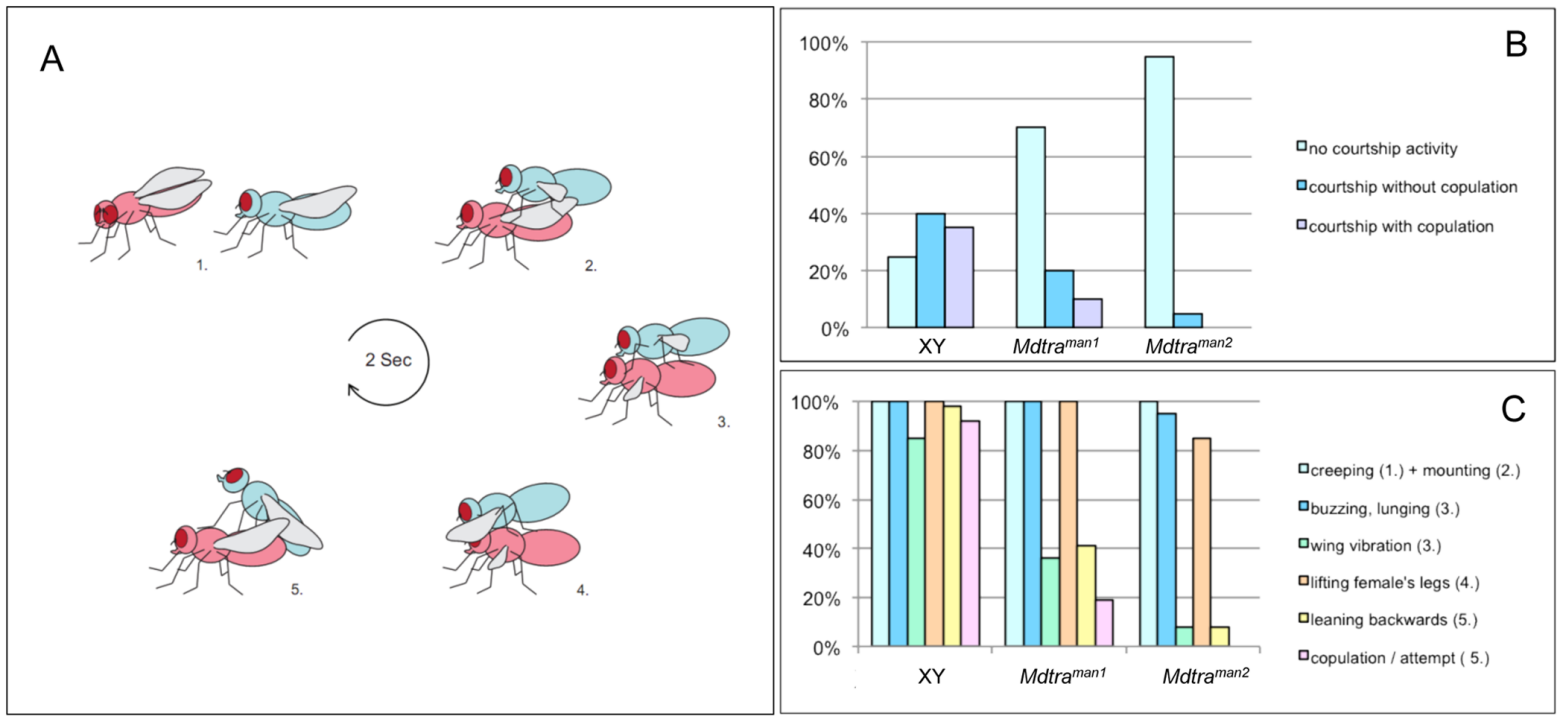
Impaired courtship performance of *Md-tra^man^* males. (A) Sketch of the housefly courtship ritual as described by [36]: (1) The male fly (blue) approaches the female (pink) and performs an abortive form of walking and stalking towards the female. The male fly will interdigitate his legs with those of the female while making tapping movements. The female may respond with similar fencing. (2) Next the male will mount the female and flutter his wings. (3) While he is lunging forward to the head of the female he will continue to flutter. He will then lift the female’s forelegs. At this point, he holds his wings in a position that is roughly 180° from their resting orientations. (4) When mounted, the female thrusts her wings 90° to her body and perpendicular to the substrate. (5) Finally, the male will lean backward and attempt to copulate. The whole ritual lasts around 2 seconds and will be repeated by the male when the first attempt fails. (B) Courtship activity of standard wild-type XY males, *Md-tra^man1^* males, and *Md-tra^man2^* males in single mating assays with wild-type females; percentage of males which courted at least once during a 20 minutes observation period (total number of males per genotype observed n  = 20). A marked reduction of courting activity is observed for *Md-tra^man1^* males and almost no activity is seen for *Md-tra^man2^* males. (C) Percentage of males performing individual steps of the courtship ritual (total number of courting males per genotype observed n  = 19). While the percentage of wildtype XY males initiating courtship remains equally high throughout the various steps of the ritual, the percentage of *Md-tra^man^* males engaged in courtship declines precipitously during the last steps, in particular step 3 (fluttering) and step 5 (copulation attempt).

Mutations affecting neuronal development can disturb behavioral outputs such as male courtship and, thus, can lead to sterility. Therefore, we decided to test courtship performance of *Md-tra^man1^* and *Md-tra^man2^* males with wild-type females. Meffert [36] described courtship in the housefly as a very brief and stereotypical ritual (see Fig. 3A). Behavioral analysis of *Md-tra^man^* mutant males showed that their overall levels of courtship were substantially lower than those of wild-type XY males (30% of *Md-tra^man1^* and 5% of *Md-tra^man2^* males compared to 80% in wild-type males) (Fig 3B). In single courtship assays, only half of the *Md-tra^man1^* males attempted copulation, while no copulation attempts were observed when *Md-tra^man2^* males were tested. In contrast, 80% of courting wild-type males attempted copulation and almost 50% successfully copulated during the 20 minutes observation period (Fig. 3B). Of the mutant males which did engage in courting, courtship latency was comparable to that of wild-type males, but copulation duration was on average three times longer than wild-type males (Table S1). To assess whether specific parts of the mating ritual were affected, we performed a second set of courtship assays using the courting scheme described by Meffert and Hagenbuch [37]. *Md-tra^man1^* and *Md-tra^man2^* males which engaged in courting performed two of the last three steps with substantially reduced frequencies, in contrast to control wild-type males which performed each step of the sequence at comparable rates (Fig. 3C and Table S2). In particular the latter steps involving wing vibrations and backward leaning of the males on top of the females are severely affected in *Md-tra^man^* males. Premature termination of the courting ritual was more often observed in *Md-tra^man2^* males than in *Md-tra^man1^* males of which 19% performed the last steps and even attempted copulation. These data suggest that the infertility observed in homozygous *Md-tra^man^* males was due to failure to properly complete the sequence of courting steps prior to copulation.

We hypothesized that residual feminizing activity of the *Md-tra^man^* allele caused male courtship failure. We tested this hypothesis by injecting dsRNA of *Md-tra* into syncytial *Md-tra^man2^* embryos. Silencing of *Md-tra* at this early stage leads to a complete and lasting loss of *Md-tra* activity [28]. Of 52 injected *Md-tra^man2^* males, 41 were fertile in single mating tests and, due to the absence of an *M* factor, produced only female offspring when outcrossed with wild-type females. The percentage of successful matings (about 80%) was comparable to those of wild-type control males in single crosses. In contrast, all 20 *Md-tra^man2^* males injected with Ringer’s buffer remained infertile. Hence, the sterility of *Md-tra^man2^* homozygous males can be rescued by targeted silencing of *Md-tra*. In a previous study we showed that silencing of *Md-tra2*, a required co-regulator for female expression of *Md-tra,* could also completely restore fertility of *Md-tra^man2^* mutant males [28], [31]. These results suggested that the residual feminizing activity of *Md-tra^man^* is sufficient to cause male courtship failure.

**Figure 4:**
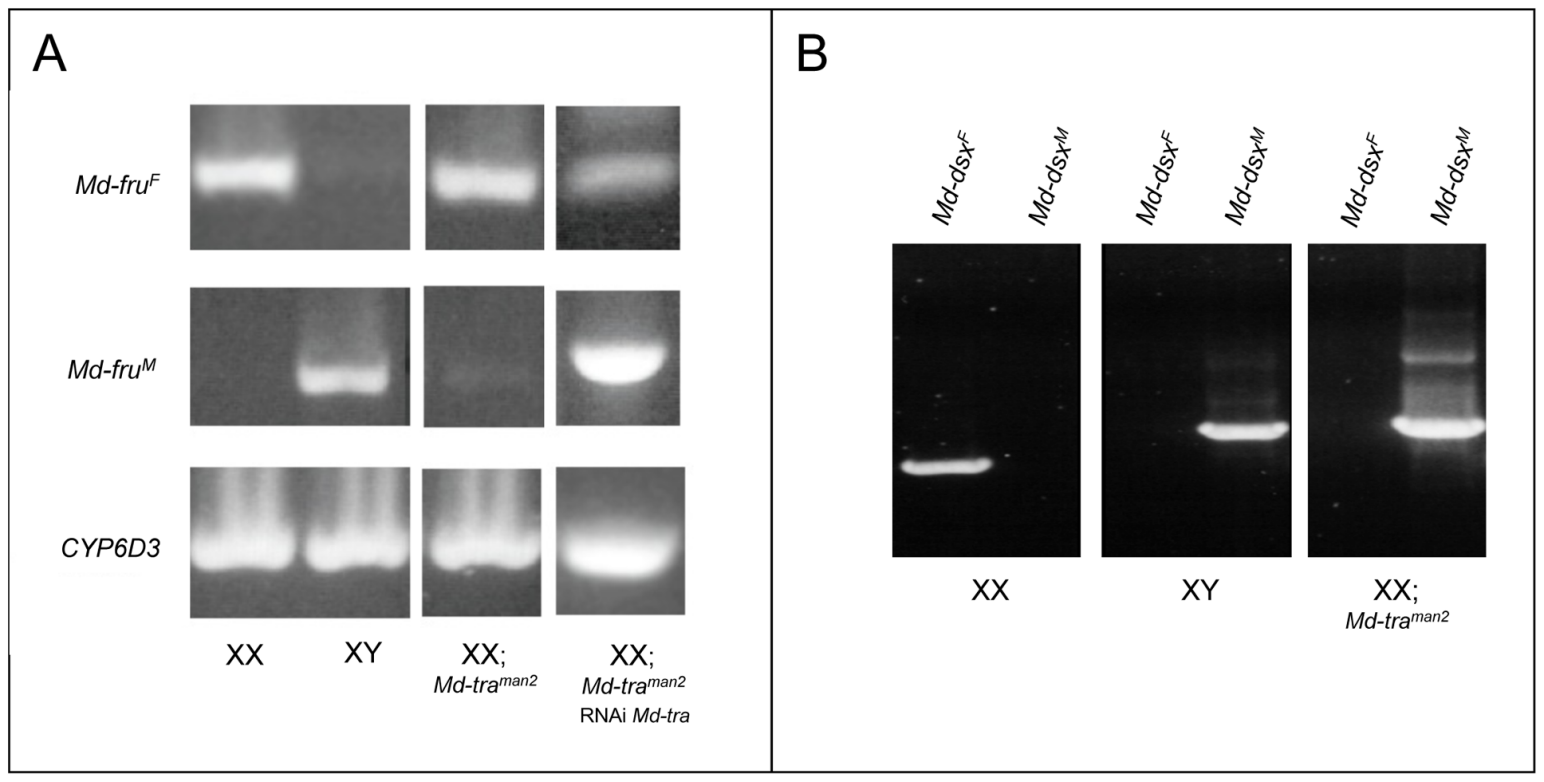
*Md-fru* but not *Md-dsx* is spliced in the female mode in *Md-tra^man2^* males. (A) *Md-fru* splicing: sex-specific primer pairs were used to detect *Md-fru^F^* transcripts (Md-fru-18 and Md-fru-1) and *Md-fru^M^* transcripts (Md-fru-27 and Md-fru-1) in wildtype XX females, in XY males, in XX; *Md-tra^man2^* males and in XX; *Md-tra^man2^* males in which *Md-tra* was silenced by embryonic dsRNA injections (RNAi *Md-tra*). As an internal control CYP6D3 transcripts were amplified from the same RNA samples. In XX; *Md-tra^man2^* males the splicing mode of *Md-fru* P1 transcripts is predominantly female whereas after silencing of *Md-tra* it shifts back into the functional male mode. (B) *Md-dsx* splicing: sex-specific primer pairs were used to detect *Md-dsx^F^* transcripts (Md-dsx-6S and Md-dsx-11AS) and *Md-dsx^M^* transcripts (Md-dsx-6S and Md-dsx-46AS) in wildtype XX females, in XY males and in XX; *Md-tra^man2^* males. Primer sequences are published in [28]. In contrast to *Md-fru*, *Md-dsx* is almost exclusively spliced in the male mode in *Md-tra^man2^* males.

This result prompted us to test whether this phenotype may be caused by misregulation of the newly identified *Md-tra* target *Md-fru*. The sex-specific splicing of *Md-fru* was investigated in *Md-tra^man2^* males. RT-PCR experiments on RNA extracted from *Md-tra^man2^* male heads detected only non-functional *Md-fru^F^* transcripts (Fig. 4A), suggesting residual *Md-tra^F^* activity in *Md-tra^man^* males is sufficient to impose female-specific splicing of *Md-fru*. In contrast, the previously described target of *Md-tra*, *Md-dsx,* seems to be unaffected as only male products were detected in *Md-tra^man2^* males (Fig. 4B). It seems likely that, in *Md-tra^man2^* males, lack of functional *Md-fru^M^* products is the primary cause of impaired courtship performance. If normal male courtship indeed depends on presence of *Md-fru^M^* we expected that male splicing of *Md-fru* must be restored in rescued *Md-tra^man2^* males. Consistent with this interpretation, we detected substantial levels of *Md-fru^M^* transcripts in rescued males (Fig. 4A) indicating a shift in the splicing mode from female back to male. We conclude that normal male behavior correlates with presence of functional *Md-fru^M^* transcripts suggesting that this gene plays a crucial role in the correct implementation of male behavior in the housefly.

### *Md-fru* is Expressed in the CNS and Peripheral Sensory Neurons

**Figure 5:**
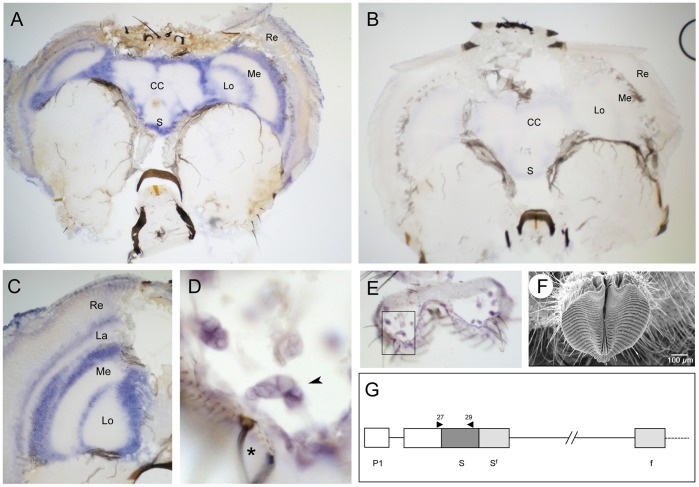
*Md-fru* P1 transcripts are expressed in the CNS and in peripheral sensory organs. (A) Frontal section of a male head hybridised with *Md-fru* P1-specific antisense RNA probe. Strong ubiquitous staining is observed in the layers below the retina and around the optic lobes (Re: Retina, La: Lamina, Me: Medulla, Lo; Lobula), as wells as around the central complex (CC) and the subesophageal ganglion (S). (B) Frontal section of a male head hybridised with the *Md-fru* P1 sense RNA probe. (C) Higher magnification of the stained areas in the optic lobes. (D) Close-up of basal neurons (arrowhead) connected to a sensory bristle (black star) located in the labellum and expressing *Md-fru* P1 transcripts. (E) Overview of the sectioned labellum shown in D (boxed). (F) SEM imaging of the fly’s mouthpart (labellum) and surrounding sensory bristles. (G). Primers used to prepare templates for P1-specific sense and antisense RNA probes are indicated as black triangles (Md-fru-27 and Md-fru-29).

In Drosophila sex-specific *fru* expression is confined to neuronal tissues [12], [20], [21], [22], [23]. To investigate the spatial expression pattern of *Md-fru* in houseflies we performed *in situ* hybridization assays. Whole mount preparations of third instar larval tissues were initially investigated. We detected ubiquitous expression of *Md-fru* P1 transcripts in both larval brain hemispheres and in the ventral ganglion. Staining was neither observed in imaginal discs nor in other non-neural tissues (data not shown). If *Md-fru* were indeed part of a developmental program involved in establishing the neuronal circuitry involved in courtship behavior, we would expect a locally restricted expression pattern in the adult CNS similar to that reported in *Drosophila*. RNA *in situ* hybridizations were performed on cryosections of adult female and male housefly heads with an antisense probe against 5′ sequences (P1-derived) of *Md-fru* (Fig. 5). Strong ubiquitous staining was detected in areas which process visual information, namely in and around the four optic neuropils lamina, medulla, lobula and lobula plate (Fig. 5A-C). Prominent *fru* expression has also been detected in the visual systems of *D. suzukii* and *D. virilis,* in particularly in the lamina, but is completely absent in *D. melanogaster* [38]. Overall, P1-specific transcripts of *Md-fru* appeared to be more broadly expressed in the mid brain than the corresponding P1 transcripts in *D. melanogaster* (Ryner et al., 1996). The *Md-fru* probe also labeled neurons located in the base of taste sensilla of the labellum (Fig. 5D–F). We propose these neurons belong to the gustatory system, as corresponding gustatory receptor neurons in *Drosophila* are known to express FRU^M^ [22].

**Figure 6:**
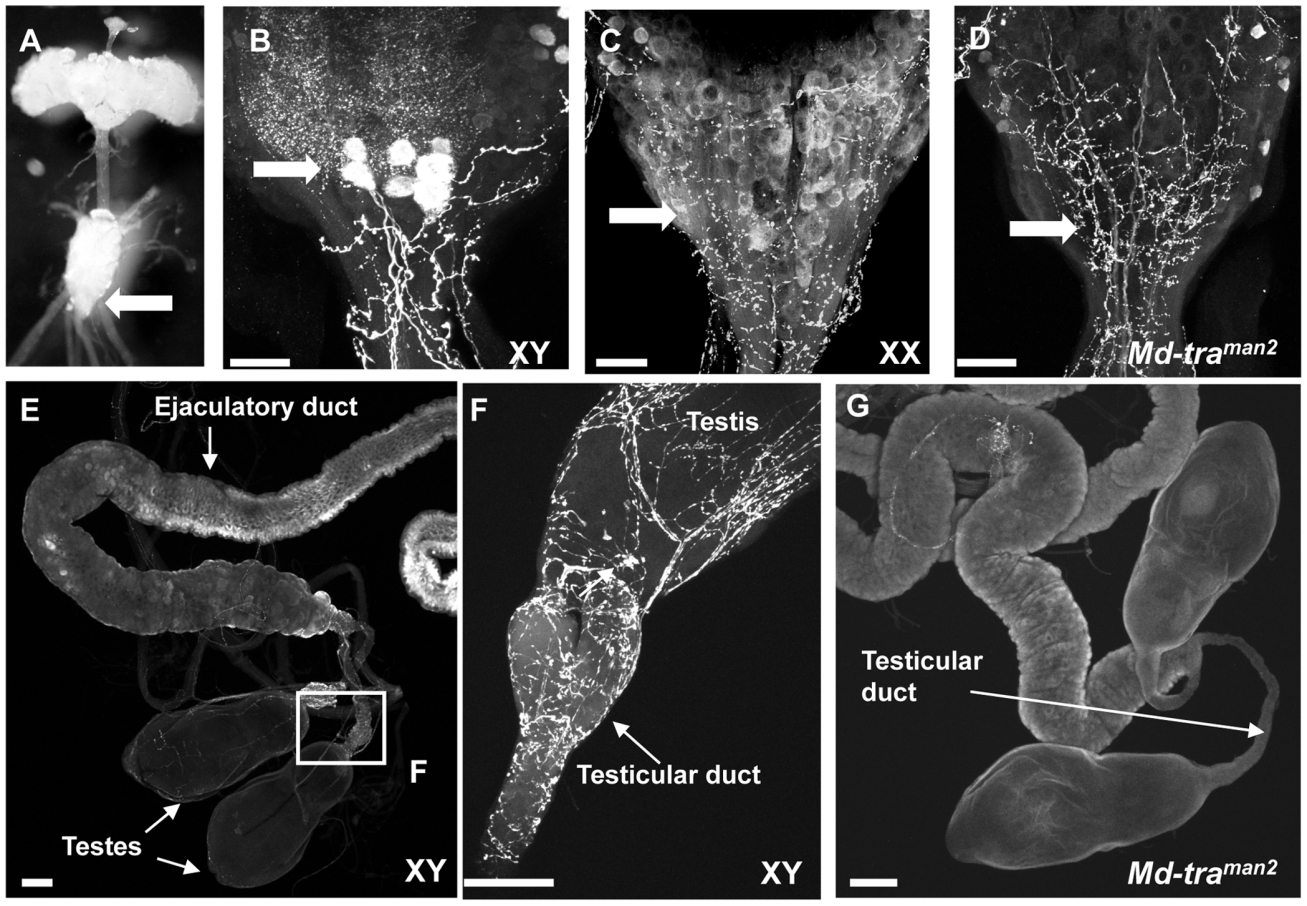
Male-specific serotonergic cluster in the ventral abdominal ganglion is absent in *Md-tra^man2^* males. (A) Whole mount CNS of an adult housefly visualized by light stereomicroscopy. The white arrow indicates the abdominal ganglion, where the SAbg cluster shown in the next panels is located. (B) Abdominal ganglion of a wild type male (XY) stained with an anti 5-HT (serotonin) antibody. The immunoreactive cell bodies of the SAbg serotonergic neurons in this region are indicated by a white arrow. (C) Abdominal ganglion of a wildtype female (XX) stained with anti 5-HT antibody. Arrow points to the corresponding area where SAbg neurons are located in males (B). (D) Abdominal ganglion of a behaviourally sterile *Mdtra^man2^* male. In contrast to wildtype males (B), there is no serotonin immunoreactivity in the corresponding area of the abdominal ganglion (white arrow). (E) Internal male reproductive organs are innervated by serotonergic projections. The white box indicates area of the testicular duct magnified in (F) showing serotonin immunoreactive varicosities. (G) Internal male reproductive organs of *Mdtra^man2^* do not show serotonin immunoreactivity. Images B-G are maximum projection of a confocal stack. Scale bars: 50 µm.

Within the sexually dimorphic neural circuitry identified in the CNS of *Drosophila melanogaster* is a cluster of 10 serotonergic neurons located in the posterior dorsal part of the abdominal ganglion which are present in males but not in females. These SAbg neurons send their projections to the main trunk that innervates the male internal reproductive organs such as the testicular ducts, seminal vesicles, accessory glands, and ejaculatory duct [39]. In *fru* mutant males these SAbg clusters are substantially diminished or completely absent suggesting that FRU^M^ is responsible for the formation and/or neurotransmitter specification of these neurons [12]. This well-documented example of *fru* function in the male CNS led us to investigate whether a similar neuroanatomical dimorphism exists in the housefly. We performed anti-5HT (serotonin) antibody staining on whole mount preparations of adult male and female CNS ventral ganglions (Fig. 6). As in *Drosophila*, we identified a small cluster of serotonergic neurons which are present at the posterior end of the abdominal ganglion in male flies, but absent in the corresponding region of the female ganglion (Fig. 6B–C). Also, projections of these serotonergic neurons are sent to the main trunk and innervate male internal reproductive organs (Figure 6E,F). Significantly, this cluster of serotonergic neurons and its projections to the male internal reproductive organs were absent in abdominal ganglions dissected from behaviourally sterile *Md-tra^man2^* mutant males (Fig. 6D,G). There was a complete lack of serotonin immunoreactivity in neurons innervating the male reproductive organs in 75% of *Md-tra^man2^* mutants (n = 12), compared with 100% immunoreactivity in wild-type controls and in *Md-tra^man1^* (n = 10 each). The loss of serotonin staining of this cluster correlates with the lack of *Md-fru^M^* expression suggesting that, just as *Drosophila*, Md-FRU^M^ plays a key role in the specification of these male serotonergic cells [13].

## Discussion

### Male Mating Behavior in Houseflies is Controlled by *Md-tra*

We previously reported that the gene *Md-tra* acts as the key switch in the sex determining hierarchy of the house fly governing every aspect of somatic dimorphic development [28]. In this study we show that courtship of male houseflies is an innate behavior that is also under the control of *Md-tra*. Our analysis of *Mdtra^man^* males revealed that infertility is primarily a result of anomalous behavior rather than of aberrant differentiation of reproductive tissues. We propose that the primary cause for anomalous courtship behavior in mutant males is the presence of female *Md-tra* activity. When *Md-tra* is knock downed by RNAi, fertility of these males is restored suggesting the absence of *Md-tra* activity is a pre-requisite for proper implementation of male courtship behavior. In females, on the other hand, *Md-tra* is activated in order to prevent the establishment of these behaviors. We propose that *Md-tra* acts as an upstream switch, coordinating dimorphic differentiation of both morphological and behavioral traits. The central role of *tra* in coordinating these two facets of dimorphic development has been extensively investigated in *Drosophila*. For instance, genotypically female XX animals which are mutant for *tra* develop into morphologically normal males which behave and court like males [40]. Reciprocally, feminization of XY individuals by activating *tra* results in the development of pseudofemales, which do not display male behaviors. Morphological and behavioral dimorphic development, however, differ with regard to the spatial and temporal requirements of *tra*. While *tra’s* functions in somatic differentiation are needed as early as during embryonic development (*e.g*., gonad differentiation), the critical developmental window for the control of sex-specific behaviors was mapped to much later stages of development around larval-pupal transition [41]. When *tra* is activated at these stages in genotypically male individuals, they will develop into normal looking males however courtship behavior is severely impaired. It suffices to target expression of *tra* to specific areas of the brain to accomplish a complete loss of courtship behavior in otherwise normally developed males [42]. This phenotype is reminiscent of the situation in *Md-tra^man^* males suggesting that residual *Md-tra* activity in these males is primarily affecting late events in dimorphic development in particular those during the critical stages when male courtship behavior is established. As feminization is only observed at the behavior level this suggests that the female activity provided by the *Md-tra^man^* alleles is either below a threshold needed to differentiate female structures or is confined to neural tissues where it specifically affects CNS related aspects of dimorphic development.

### *Md-fru* is a Target of the Sex-determining Pathway Downstream of *Md-tra*

In this study we have identified the house fly homologue of the key *Drosophila* sex-determination gene *fru*, *Md-fru*. We have shown that, in an analogous manner to *Drosophila*, *Md-fru* RNAs are alternatively spliced leading to male-specific expression of Md-FRU^M^ proteins from the P1 promoter. The presence of four putative TRA/TRA2 bindings sites located close to the regulated splice sites of the female-specific exons of *Md-fru* suggests that this regulation is due to its targeting by the female-specific splicing factor *Md-tra*. This is similar to the regulation of the previously proposed targets of *Md-tra*, *Md-dsx* and *Md-tra* itself [28], [30]. We show that changes in *Md-tra* activity lead to corresponding changes in *Md-fru* expression. For instance, ablating *Md-tra* function by RNAi in karyotypically female individuals shifts the mode of *Md-fru* splicing from female to male. Reciprocally, when *Md-tra* functions are ectopically expressed in karyotypic males, *Md-fru* expression switches to the female nonfunctional mode. We additionally show that *Md-tra2,* a functional splicing co-factor with *tra* in *Drosophila*, is involved in *Md-fru* splicing regulation, by silencing *Md-tra2* in *Md-tra^man2^* males and showing a shift of *Md-fru* splicing from the female to the male mode (data not shown) [31].

Taken together we propose that, like *Md-dsx* and *Md-tra*, *Md-fru* is another downstream and possibly direct target of *Md-tra* in the house fly sex determination pathway. In *D. melanogaster*, male-specific FRU^M^ products are required for the proper display of male courtship behavior. When *tra* is ectopically activated in the developing male CNS, it prevents the production of FRU^M^ and, as a consequence, these males exhibit impaired and anomalous courtship [3], [43], [44]. Along the same lines, we propose that *Md-fru* is a target of *Md-tra* and that misregulation of *Md-fru* by residual *Md-tra* activity is the primary cause for courtship failure of *Md-tra^man^* males. Interestingly, the sex-specific splicing of *Md-fru*, and not *Md-dsx*, seems to be affected by the hypomorphic activity produced by the *Md-tra^man^* alleles. In *Md-tra^man^* mutant males the splicing of *Md-fru* shifts from the male to female mode, resulting in the loss of ability to produce male-specific FRU proteins.

The disparity in splicing behavior of *Md-dsx* and *Md-fru* in the hypomorphic *Md-tra^man^* background can be explained by either different thresholds requirements or by differences in temporal and spatial requirements for activating the female splicing mode. For instance, *Md-fru* pre-mRNAs may have a higher affinity for Md-TRA binding than *Md-dsx* pre-mRNAs or may require lower levels of bound complexes to activate the female-specific splice sites. On the other hand, the differential effects on *Md-dsx* and *Md-fru* may result from discontinuous and/or tissue-specific *Md-tra^man^* expression during development. In either case, its prominent impact on behavioral dimorphic development suggests that *Md-tra^man^* must be expressed in the nervous system around the time when the CNS shapes the neuronal circuits required for proper male behavior [41]. Consistent with this interpretation, we detected female *fru* transcripts (*Md-fru^F^*) in the heads of male *Md-tra^man^* adults. We propose that *Md-fru* is a direct target of *Md-tra* thereby defining an *Md-dsx* independent branch in the sex-determining pathway, which specifies the fates or activities of neurons that carry out higher order control functions to elicit and coordinate the activities comprising male reproductive behavior.

### Is *Md-fru* Functionally Conserved?

We propose that male-specific *Md-fru^M^* expression is required for the correct display of male courtship in the housefly. This conclusion is based on the strict correlation between proper courtship performance and male expression of *Md-fru*. In our courtship assays we found that only males that expressed the *Md-fru^M^* variants performed as well as normal males, while those that expressed non-functional *Md-fru^F^* variants either performed poorly or did not engage in courtship at all. The most compelling argument comes from the observation that silencing of *Md-tra* in *Md-tra^man2^* males not only shifts *Md-fru* expression from the female to male mode but also completely restores fertility. A more direct test to confirm the role of *Md-fru* in courtship behavior would require a targeted disruption of *Md-fru^M^* function (*cf*. [8]). We expect that silencing of *Md-fru^M^* causes behavioral phenotypes similar to those observed in *Md-tra^man^* males. Unfortunately, several attempts to specifically disrupt *Md-fru^M^* functions have thus far failed. Injections of dsRNA against P1 derived sequences of *Md-fru* into early syncytial blastoderm embryos failed to downregulate *Md-fru* expression in larval and pupal stages (data not shown). To overcome this problem we introduced a construct into the housefly genome which contains inverted repeats against P1 derived *Md-fru* transcripts driven by a constitutively expressed promoter. Unfortunately, none of the tested transgenic lines effectively suppressed *Md-fru* (data not shown). On the other hand, downregulation of the respective *fru* homolog in the hemimetabolous species, *Blatetella germanica* and *Schistocerca gregaria*, both of which are responsive to systemic effects of dsRNA silencing, have been shown to disrupt proper male behavior suggesting that *fru* plays an important and widely conserved role in male behavior [45], [46], [47]. However, it is not known whether the *fru* homologs in these species are direct targets of the sex-determining cascade and whether of not these homologs are specifically expressed in the CNS.

The functions of *fru* in male courtship have been studied in great detail at the cellular level in *Drosophila*. FRU^M^ is expressed in about 1700 neurons which appear to be involved in various aspects of male courtship including olfactory and gustatory neurons required for behavioral responses to sex pheromones. Likewise, we observe that expression of *Md-fru* is confined to specific areas of the CNS and to neurons of the peripheral nervous system. Though expression appears much broader than in *Drosophila* and apparent in the optic neuropils, its neural-specific expression suggests that *Md-fru* is also involved in building and specifying neuronal circuits required for male behavior. High levels of expression in the neuropils which process visual cues hint at a prevalent role of the visual system in male behavior of the housefly. Previous studies showed that visually guided behavior patterns in houseflies differ between the sexes. Male houseflies chase females in the air using visual cues, but are not chased by females [48], coinciding with this sex-specific behavior are the presence of male-specific neurons in the visual system [49], [50]. It is thus possible that expression of *Md-fru* in the optic neuropils is involved in establishing the sexual difference of neural architecture involved in motion detection.

One striking dimorphic neural trait common to houseflies and fruit flies is the presence of a male-specific cluster of serotonergic neurons in the dorsal posterior part of the abdominal ganglion. In both types of dipterans these neurons send projections to the male internal reproductive organs where they innervate contractile muscles. It has been proposed that these muscles are required for transferring sperm and seminal fluids during copulation [51], [52]. In semi-fertile *fru* mutant males this cluster is substantially reduced in size and projection patterns are aberrant [51]. A similar phenotype was found in *Md-tra^man2^* housefly males where the cluster is substantially diminished in size and projections to the internal reproductive organs are lost. In *Drosophila* the formation and/or specification of the male-specific serotonergic cluster depends on presence of FRU^M^ and DSX^M^ activity [12], [21]. It is thus conceivable that lack of Md-FRU^M^ is the cause for their absence in *Md-tra^man2^* males, suggesting a conserved mechanism in the specification of these male-specific neurons.

**Figure 7:**
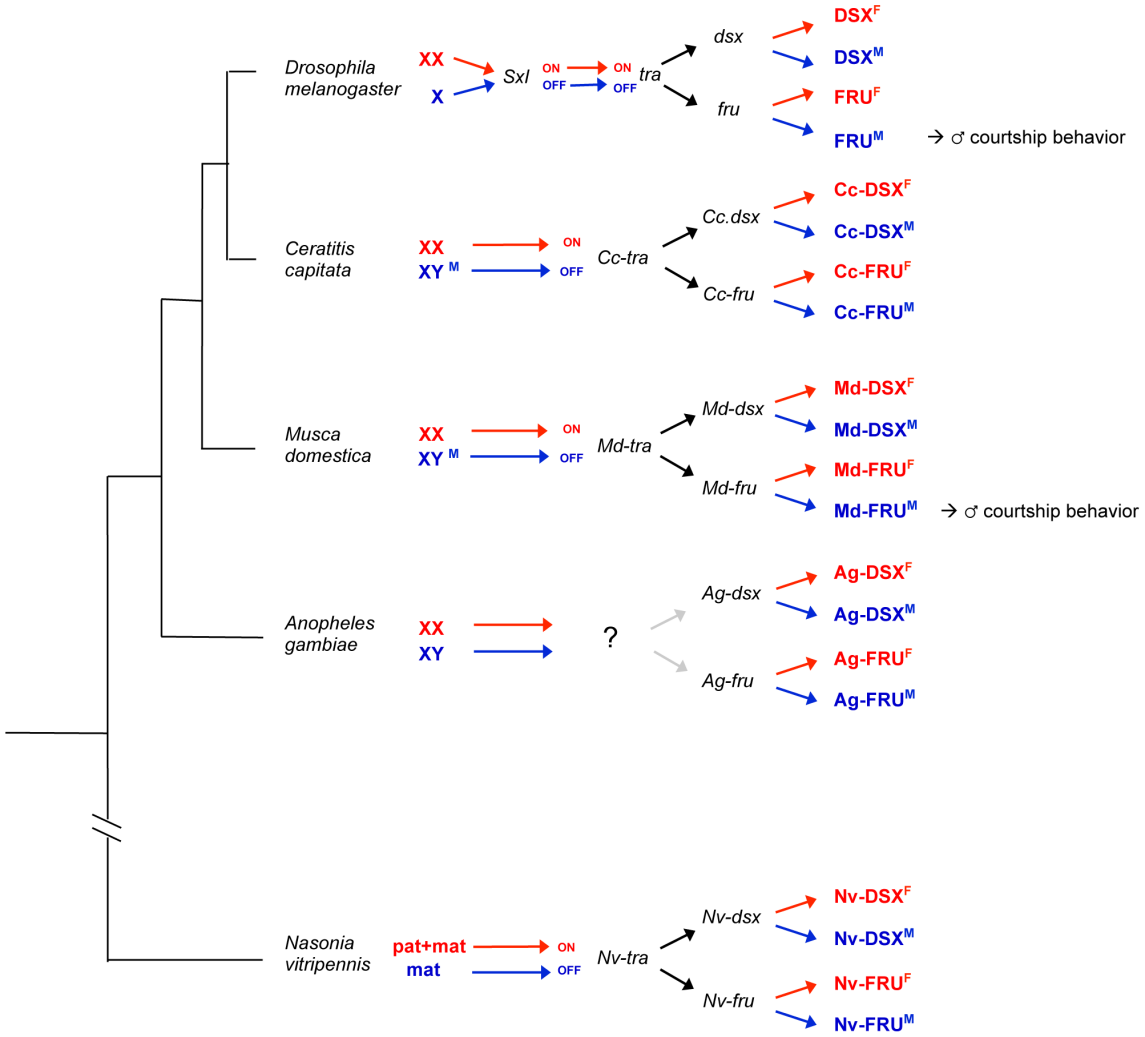
Bifurcation of the sex-determination pathway: a general principle in holometabolous insects? The phylogenetic relationship and the sex-determining pathways of several dipteran species and the hymenopteran species Nasonia are depicted in this scheme. Despite considerable differences regarding the nature of the instructive sex-determining signal (X-counting in Drosophila, male [M] dominant determiners in Musca and Ceratitis, haplo-maternal [mat] versus diplo-maternal-paternal [pat+mat] in Nasonia) many insects use *tra* as the binary on/off switch downstream of the signal [54], though the molecular basis of this switch in *A. gambiae* remains still elusive [24], [55], [56]. The *dsx* gene, expressing sex-specific variants DSX^F^ and DSX^M^, appears to be a conserved target of the binary switch and its use as a bifunctional executor of the instructive signal was validated by gene function studies in the depicted insect species [26], [30], [56], [57], [58] Also, homologs of *fru* were identified in these species that express sex-specific variants FRU^F^ and FRU^M^ [24], [26], [27], [59]. For Musca we here present evidence that *fru* is a downstream target of the *tra* branch which is involved in controlling proper display of courtship. Though no functional *fru* data from other holometabolous insects are available as yet, we propose that *fru* independently from *dsx* represents another conserved effector of the instructive signal. Hence, bifurcation of the pathway downstream of *tra* may have already existed before the divergence of holometabolous insect orders.

Our study in the housefly suggests that merging the control of the two terminal pathway effectors, *fru* and *dsx*, at the level of *tra* is a widely used strategy in holometabolous insects (Fig. 7). We propose that this linked control ensures that sex-determining instructions are coordinately relayed and implemented in neural and non-neural tissues.

## Materials and Methods

### Rearing of the Flies

Rearing of *Musca domestica* larvae and flies has been described previously [33]. Since low density of larvae on standard medium can cause substantial decrease in survival rates, we reared transplanted host larvae and larvae from injected embryos on porcine manure. To dispose of mites and other parasites and to avoid contamination with eggs or larvae from wild**-**type populations, manure was stored at −70°C for at least two weeks prior to use.

### Strains of *Musca Domestica*

(1) Wild-type strain: females *XX*, males *XY*; (2) autosomal *M* strain: females *XX; pw, bwb/pw, bwb*, males *XX; M^III^, pw^+^, bwb^+^/pw, bwb*; (3) *Md-tra^man1^* strain: females *XX, Md-tra^man^/Md-tra^+^*, males *XX, Md-tra^man^/Md-tra^man^* [33]; (4) *Md-tra^man2^* strain: females *XX; Mdtra^D^, Ba/Mdtra^man^, Ba^+^*, males *XX; Mdtra^man^, Ba^+^/Mdtra^man^, Ba^+^*; (5) multimarked strain: females *XX; ac/ac; ar/ar; bwb/bwb; ye/ye; snp/snp*, males *XY; ac/ac; ar/ar; bwb/bwb; ye/ye; snp/snp*.

### Transplantation of Larval Gonads

Crossing schemes and used genotypes are shown in Fig. S2. Larval anlagen of *Mdtra^man2^, bwb* ^+^ testes were dissected and transplanted into the posterior region of *bwb* male host larvae in Ringer’s solution. These larvae were then reared on porcine manure and surviving adults were outcrossed with *bwb* females of the host strain. In the progeny we screened for presence of donor derived *bwb* ^+^ individuals.

### Sperm Analysis

Testes of 5-days old adult males were dissected in Ringer’s solution and transferred to a coverslip where they were gently squeezed to release sperm which was then dispersed with a needle. Morphology and mobility of sperm were examined by phase-contrast microscopy.

### Courtship Assays

Male and female flies collected for courtship assays were separated shortly after hatching and kept at 25°C for 6 days in plastic beakers with sugar water and milk powder. The air humidity was kept between 65% and 75% and flies were exposed to a 12 hours light/dark cycle. The courtship assays were performed at the same time of the day. The courtship arena consisted of a see-through plastic cylinder with a height of 40 mm and a length of 160 mm. For each couple courtship activities were examined during a period of at least 20 minutes.

### *Md-tra* dsRNA Injections

The same primers were used for synthesis of *Md-tra* dsRNA as described in [28]: *Md-tra* forward primer located in the 5′UTR of exon 1 linked to T7 promoter sequences (small letters): (5′ gtaatacgactcactatagggTGGTGTAATATGGCTCTATCG 3′) and *Md-tra* reverse primer located in exon 5 linked to T7 promoter sequences (small letters): (5′ gtaatacgactcactatagggGCTGCCATACAAACGTGTC 3′).

Total RNA from adult females was reverse transcribed and the resulting cDNA was amplified with this primer pair to generate a female-specific template of 620 bp in length. In vitro transcription of this template with T7 RNA polymerase yielded dsRNA that was ethanol precipitated and resuspended in injection buffer to a final concentration of 1 µg/µl. Embryos were collected within 1 h after egg laying and dechorionated. Then 1–2 nl of the dsRNA solution was injected into the posterior pole of syncytial blastoderm embryos [28]. As a negative control, embryos of the same stage were injected with a similar volume of Ringer’s solution. Injected embryos were allowed to develop at room temperature and placed in porcine manure for further development. Hatching flies were mated to virgins of the multimarked strain.

### Isolation of *Md-fru*

A small *Md-fru* fragment spanning the conserved BTB domain was previously recovered by David Dolezel (University of Ceske Budejovice, Czech Republic) using degenerated primers and made available to us for further investigation. Based on sequence alignments between *fru* sequences of *Drosophila* and *Anopheles gambiae* [24] we generated degenerated primers for the different zinc finger-domains:

domain A: Md-fru-6 (5′ RCAATGNGCYTTCATRTTATC 3′),

domain B: Md-fru-7 (5′ ATGNSWATGRAAATGATGCCA 3′),

domain C: Md-fru-8 (5′ CATNGGATGYTTRAAYTTRCA 3′).

These reverse primers were used in combination with the forward primer MdFRU-3E (5′CAGTTAAGGCTCATCAAGCC3′) located in the BTB domain to amplify cDNA variants containing zincfinger domains B and C. Forward primer MdFRU-15s (5′CATGGGCCTCAATCAGTCG3′) was used to amplify the variant which contains zinc finger domain A. The zinc finger domain D was identified by bioinformatics in the genomic region between exon C4 and exon C5.

### 5′ and 3′ RACE of *Md-fru* cDNA

Female and male heads were collected from freshly hatched adults and total RNA was prepared according to the protocol of the manufacturer TRI REAGENT (SIGMA-ALDRICH, St Louis, USA) or to the protocol of the “NucleoSpin RNAII” kit (Macherey-Nagel, Düren, Germany). For long term storage, total RNA was dissolved in 70% ethanol and stored at −80°C.

3′ RACE was performed according to the protocol “5′/3′ RACE Kit, 2^nd^ Generation” (Roche Applied Sciences, Rotkreuz, Switzerland). For 3′ RACE, the supplied oligo-dT primers were used for reverse strand cDNA synthesis. Forward primers located in the zincfinger encoding exons A, B, and C, were used for second strand synthesis and subsequent amplification: Md-fru-70B in domain A (5′ GCGATGACTTGAGTCTGACA 3′), Md-fru-35 in domain B (5′ CAACCTGAGGACACATTGCA 3′), Md-fru-36 in domain C (5′ GGACGTATGTCAAGAACCTC 3′) in combination with the shortened PCR-anchor primer A-3 (5′ ACGCGTATCGATGTCGAC 3′). For 5′ RACEs, gene-specific primer Md-fru-1 (5′ TCTGAGTATCTGACATCCTTC 3′) was used for reverse transcription. First round PCR amplification used gene-specific primer Md-fru-4E (5′ GCAGGCTAGAGTAACATCAC 3′) and the oligo-dT primer supplied with the Kit. Second round PCR amplification used gene-specific primer Md-fru-23 (5′ GACACCGGTTAAATTTGTCGG 3′) and a shortened PCR-anchor primer A-3 provided by the manufacturer Boehringer. In the first round PCR, the polymerase and Buffer 3 of the “Expand Long Template PCR System” kit by the manufacturer Roche Diagnostics (Risch, Switzerland) was used. For the second round PCR, the “*Taq* DNA Polymerase in Storage Buffer B” by the manufacturer Promega (Madison, USA) was used.

### RT-PCR Analysis of Sex-specific *Md-fru* Splice Variants

The following primers were used to monitor amplify the sex-specific splice variants of *Md-fru* transcripts. Forward primers: Md-fru-18 (5′ TCCATCATGAGCGAACTCTGT 3′) is located in the female-specific sequences of the P1 transcripts, Md-fru-27 (5′ GTGAAGTGAACTTAGTACATGG 3′) is located in the common sequences of the P1 transcripts; reverse primer: Md-fru-1 (5′ TCTGAGTATCTGACATCCTTC 3′), located in the C1 exon. As an internal control the cytochromatic gene *CYP6D3* was amplified with the following primers: CYP6D3-1 (5′ GTTCGGTAATATTTGGCTTGG 3′) and CYP6D3-2 (5′ CCCGTATTCCGTAGTTGAATT 3′). Primer pair Md-fru-18 and Md-fru-1 yields a fragment of 289 bp in females, and primer pair Md-fru-27 and Md-fru-1 generates a 602 bp long PCR product in males.

When testing the splicing mode of *Md-fru* in *Mdtra* RNAi treated XX males, the following primer pair was used for reamplification: Md-fru-2 (5′ TCGAAATATGGACTGCATGCTG 3′; in exon C2) and Mdfru-28 (5′ GTTTGTTCTCTGTCCAGTGTT 3′; in the common part of exon P1).

### Multiple Sequence Alignments

For multiple sequence alignments the ClustalW algorithm provided by EMBL (http://www.ebi.ac.uk/Tools/msa/clustalw2/) was used.

### *In situ* Hybridisation of *Md-fru*P1 Transcripts on Adult Head Sections

Probes were derived from templates generated by PCR with primer Md-fru-27 (forward; 5′ GTGAAGTGAACTTAGTACATGG 3′) and Md-fru-29 (reverse; 5′ CTGTCAAGGGTTCTCGGCTT 3′): A T7 promoter sequence (GTAATACGACTCACTATAGGG) was added to the Md-fru-27 primer for preparing sense probes (control) and to the Md-fru-29 primer for preparing antisense probes. Both amplicons were transcribed with T7 polymerase to generate labelled single stranded RNA probes (Roche, Madison, USA). Heads were dissected from adult flies, mounted in O.C.T medium and frozen at −20°C prior cryosectioning with a Leica rotary microtome. *In situ* hybridizations were carried out on adult head sections according to the protocol of [53].

### Serotonin Staining of Abdominal Ganglions

Abdominal ganglions were dissected in PBS solution and fixed in 4% paraformaldehyde in PBS for 1 hour on ice while gently shaking. Then the tissues were washed three times in PBS for 15 minutes each. Following a brief 5 minutes incubation in 1 N HCl, the tissues were washed in PBS for 15 minutes and three times in 0.4% PBT for 10 minutes each. Afterwards the tissue samples were incubated for 1 hour in PTN (0.4% PBT, 5% (v/v) normal goat serum), followed by the incubation with the primary antibody for 24–36 hours at 4°C. The rabbit anti-serotonin (5-HT) antibody was purchased from SIGMA-ALDRICH (cat # S5545). Dilution of the antibody was 1/400 in PTN. After the incubation of the primary antibody the ganglions were washed four times in PBT for 30 minutes each, followed by the incubation of the secondary antibody at 4°C overnight or at room temperature for 3 to 4 hours. Dilution of the secondary antibody (anti-rabbit FITC, SIGMA-ALDRICH, anti-rabbit AlexaFluor 488, Molecular Probes Eugene, USA) was 1/600 in PTN. To remove unbound secondary antibody the samples were washed four times in PBT for 1 hour each. Thereafter, the samples were washed several times in PBS for several hours each (1–2 days in total). Finally, the samples were mounted in VectaShield medium (Vector Labs, Burlingame, USA) on glass slides and covered with cover slips. The mounted samples were examined and analysed by confocal laser scanning microscopy (Zeiss LSM 510). The genotypes of all animals were coded before dissections to avoid bias in either preparation or scoring of the samples.

## Supporting Information

Figure S1 **Amino acid sequence alignments of** ***fru*** **homologs in different dipterans and in Tribolium and Nasonia. (A)** Alignments of the male-specific N-terminal extension of *fru* in *M.domestica*, *D. melanogaster*, *A. gambiae*, *N. vitripennis*. The putative start codon is labelled in bold. **(B)** Alignment of the BTB domain of *fru* homologs from the same species as in (A) with the addition of *in silico* identified *fru* sequences of *T. castaneum*. The BTB domain overall shows a high degree of sequence similarity. **(C)** Alignment of the zinc-finger A encoding exon. **(D)** Alignment of the zinc-finger B encoding exon. **(E)** Alignment of the zinc-finger C encoding exon. **(F)** Alignment of the zinc-finger D encoding exons. Identical amino acids are indicated with an asterisk (*). Highly similar amino acids are labelled with a colon (:) while amino acids with weak similarity are indicated with a dot (.). Similarity scores between the different domains of Musca and Drosophila *fru* are: 54% (A), 98% (B), 90% (C), 74% (D), 54% (E) and 54% (F).
(TIF)

Figure S2 **Larval testes transplantations.** Male *Md-tra^man2^* progeny (donor) was generated by crossing *bwb* ^+^; *Md-tra^man^* males with *bwb* ^+^; *Md-tra^man^*/*Md-tra^D^* females. Gonads were dissected from these male larvae and transplanted into male *bwb*, *M^III^*; *Md-tra^+^* larvae (host). These larvae were reared to adulthood and outcrossed with females of the host strain. Three different phenotypes of progeny were expected, *bwb* males and females (light brown) from host derived sperm and *bwb* ^+^ females (dark brown) from donor derived sperm. We recovered 7 successfully transplanted males which sired in total 728 individuals with a host genotype (*bwb*) and 154 individuals with a donor genotype (*bwb* ^+^).
(TIF)

Table S1 **Courtship latency and duration of wild-type males and mutant** ***Md-tra^man^*** **males.**
(DOCX)

Table S2 **Frequencies of discrete courtship elements observed in courting wild-type males and** ***Md-tra^man^*** **males.**
(DOCX)
