# Supplementary figures and images for: Genetic Control of Courtship Behavior in the Housefly: Evidence for a Conserved Bifurcation of the Sex-Determining Pathway
Source: PLoS One. 2013 Apr 22;8(4):e62476. doi: 10.1371/journal.pone.0062476 (PMC3632534; doi:10.1371/journal.pone.0062476)

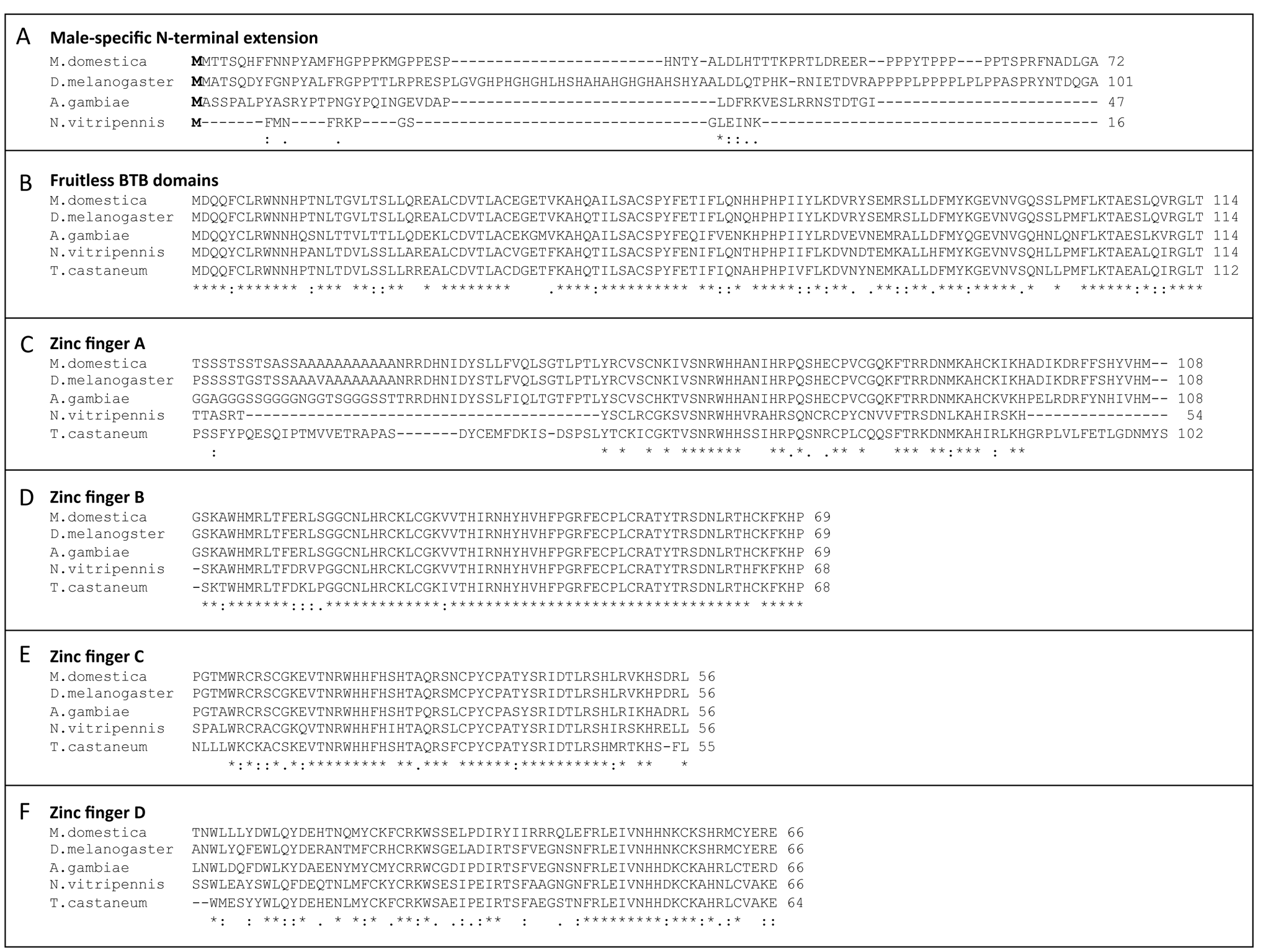

Supplement: Figure S1 — Amino acid sequence alignments of fru homologs in different dipterans and in Tribolium and Nasonia. (A) Alignments of the male-specific N-terminal extension of fru in M.domestica, D. melanogaster, A. gambiae, N. vitripennis. The putative start codon is labelled in bold. (B) Alignment of the BTB domain of fru homologs from the same species as in (A) with the addition of in silico identified fru sequences of T. castaneum. The BTB domain overall shows a high degree of sequence similarity. (C) Alignment of the zinc-finger A encoding exon. (D) Alignment of the zinc-finger B encoding exon. (E) Alignment of the zinc-finger C encoding exon. (F) Alignment of the zinc-finger D encoding exons. Identical amino acids are indicated with an asterisk (*). Highly similar amino acids are labelled with a colon (:) while amino acids with weak similarity are indicated with a dot (.). Similarity scores between the different domains of Musca and Drosophila fru are: 54% (A), 98% (B), 90% (C), 74% (D), 54% (E) and 54% (F). (TIF) [file pone.0062476.s001.tif]

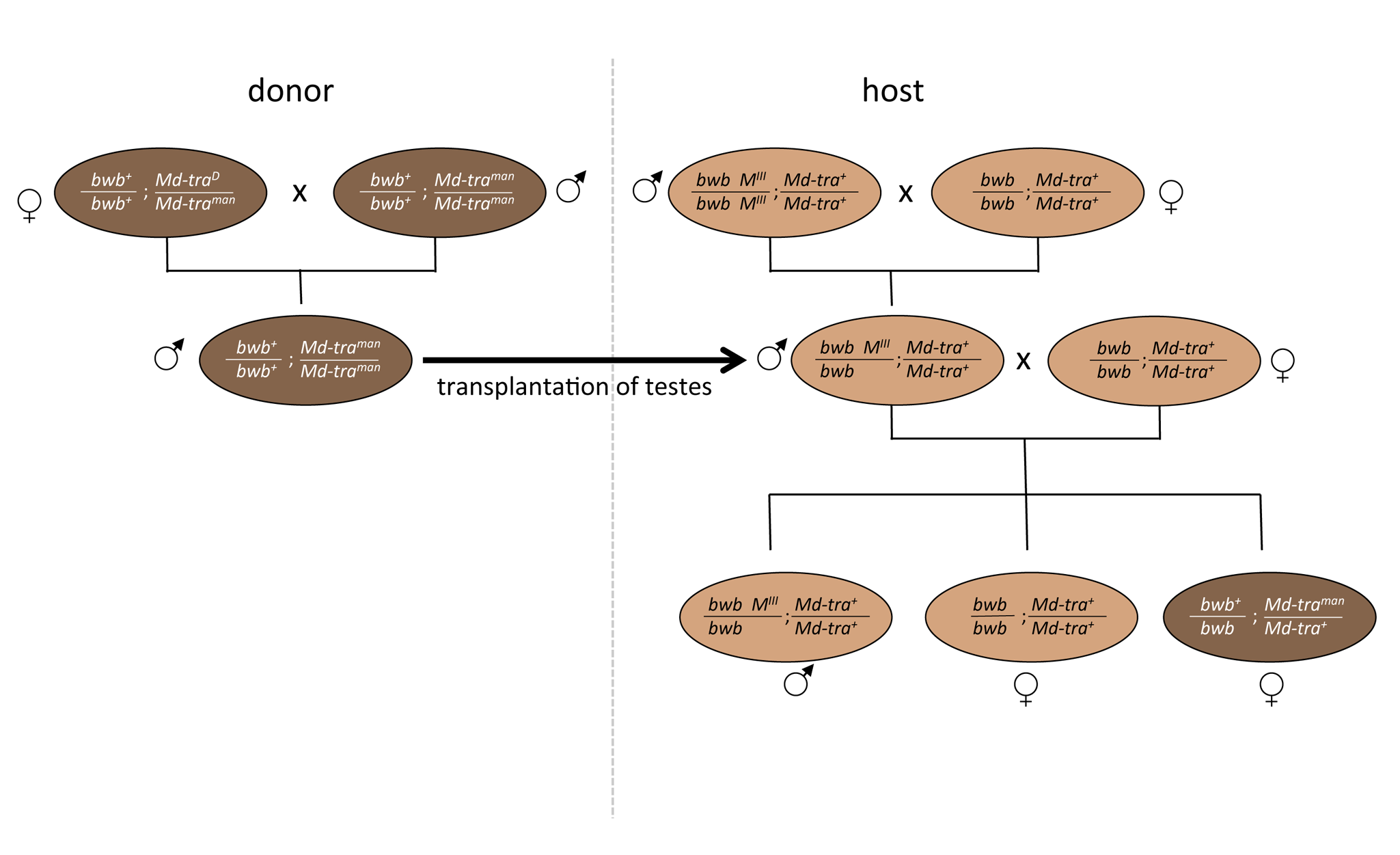

Supplement: Figure S2 — Larval testes transplantations. Male Md-traman2 progeny (donor) was generated by crossing bwb +; Md-traman males with bwb +; Md-traman/Md-traD females. Gonads were dissected from these male larvae and transplanted into male bwb, MIII; Md-tra+ larvae (host). These larvae were reared to adulthood and outcrossed with females of the host strain. Three different phenotypes of progeny were expected, bwb males and females (light brown) from host derived sperm and bwb + females (dark brown) from donor derived sperm. We recovered 7 successfully transplanted males which sired in total 728 individuals with a host genotype (bwb) and 154 individuals with a donor genotype (bwb +). (TIF) [file pone.0062476.s002.tif]
