# Supplementary material for: Genetic Control of Courtship Behavior in the Housefly: Evidence for a Conserved Bifurcation of the Sex-Determining Pathway
Source: PLoS One. 2013 Apr 22;8(4):e62476. doi: 10.1371/journal.pone.0062476 (PMC3632534; doi:10.1371/journal.pone.0062476)
Supplement: Table S1 — Courtship latency and duration of wild-type males and mutant Md-traman males. (DOCX) [file pone.0062476.s003.docx]

Table S1 Courtship latency and duration of wild-type males and mutant *Md-tra^man^* males

|  | XY standard males | | *Md-tra^man1^* males | | *Md-tra^man2^* males | |
| --- | --- | --- | --- | --- | --- | --- |
|  | **no** copulation ^a^ | copulation | **no** copulation | copulation | **no** copulation | copulation |
| courting males (n=20) | 8 (40%) | 7 (35%) | 4 (20%) | 2 (10%) | 1(5%) | 0 |
| Δt until csb ^b^ | 6'07'' (15''- 19'06'') | 7'24'' (29'' - 17'46'') | 6'21'' (2'00'' - 10'39'') | 7'26'' (1'51'' - 13'01'') | 19'28'' |  |
| # of csbs per male ^c^ | 4.4 (1 -15) | 2.1 (1 - 5) | 9.75 (1 -29) | 5.5 (1 -10) | 1 |  |
| duration of csb | 17'' (1'' - 3'59'') | 5'' (1'' - 12'') | 17'' (1'' - 2'10'') | 17'' (1'' - 2'10'') | 1'' |  |
| Δt until copulation |  | 9'18'' (29'' - 18'49'') |  | 14'45'' (13'01'' - 16'29'') |  |  |
| Δt of copulation |  | 89' (67' - 155') |  | 64' (56' - 72') |  |  |

^a^ males which started courting but did not copulate

^b^ average latency time until csb (courtship behavior) starts. In parentheses observed time range is indicated

^c^ average number of csbs (courtship behaviors) per male. Range is indicated in parentheses
