# Supplementary material for: Genetic Control of Courtship Behavior in the Housefly: Evidence for a Conserved Bifurcation of the Sex-Determining Pathway
Source: PLoS One. 2013 Apr 22;8(4):e62476. doi: 10.1371/journal.pone.0062476 (PMC3632534; doi:10.1371/journal.pone.0062476)
Supplement: Table S2 — Frequencies of discrete courtship elements observed in courting wild-type males and Md-traman males. (DOCX) [file pone.0062476.s004.docx]

Table S2 Frequencies of discrete courtship elements observed in courting wild-type males and *Md-tra^man^* males

|  | XY standard males (n=19) | | | *Md-tra^man1^* males (n=19) | | | *Md-tra^man2^* males (n=19) | | |
| --- | --- | --- | --- | --- | --- | --- | --- | --- | --- |
|  | total n ^a^ | mean^b^ | SD^c^ | total n | mean | SD | total n | mean | SD |
| creeping & moving forward | 40 | 2,11 | 1,37 | 43 | 2,26 | 1,58 | 28 | 1,47 | 0,82 |
| lunging | 40 | 2,11 | 1,37 | 43 | 2,26 | 1,58 | 27 | 1,42 | 0,88 |
| wing vibration | 35 | 1,84 | 1,56 | 14 | 0,74 | 0,96 | 2 | 0,11 | 0,31 |
| tapping | 40 | 2,11 | 1,37 | 43 | 2,26 | 1,58 | 25 | 1,32 | 0,98 |
| leaning backward | 39 | 2,05 | 1,39 | 15 | 0,79 | 1,28 | 1 | 0,05 | 0,22 |
| copulation / attempt | 36 | 1,89 | 1,33 | 5 | 0,26 | 0,55 | 0 | 0 | 0 |

^a^ total number of displayed element per genotype

^b^ mean number of element display per male

^c^ standard deviation
